# Targeted Cellular Treatment of Systemic Lupus Erythematosus

**DOI:** 10.3390/cells14030210

**Published:** 2025-01-31

**Authors:** Panagiotis Athanassiou, Lambros Athanassiou, Ifigenia Kostoglou-Athanassiou, Yehuda Shoenfeld

**Affiliations:** 1 Department of Rheumatology, St. Paul’s Hospital, 55134 Thessaloniki, Greece; 2 Department of Rheumatology, Asclepeion Hospital, Voula, 16673 Athens, Greece; lambros.ath@gmail.com; 3 Department of Endocrinology, Asclepeion Hospital, Voula, 16673 Athens, Greece; ikostoglouathanassiou@yahoo.gr; 4 Zabludowicz Center for Autoimmune Diseases, Sheba Medical Center, Reichman University, Herzliya 4610101, Israel; yehuda.shoenfeld@sheba.health.gov.il

**Keywords:** systemic lupus erythematosus, B lymphocyte, stem cell transplantation, mesenchymal cell transplantation, CAR T cell therapy

## Abstract

Systemic lupus erythematosus (SLE) is a systemic autoimmune disease affecting all organ systems. The disease preferentially affects females of childbearing age. It runs a variable course. It may run a mild course that may never lead to severe disease and manifestations from critical organ systems. However, it may also run an undulating course with periods of mild and severe disease. It may run as a mild disease, quickly deteriorating to severe disease and affecting multiple organ systems. Various immune pathways related both to the innate and adaptive immune response are involved in the pathogenesis of SLE. Various drugs have been developed targeting cellular and molecular targets in these pathways. Interferons are involved in the pathogenesis of SLE, and various drugs have been developed to target this pathway. T and B lymphocytes are involved in the pathophysiology of SLE. Various treatment modalities targeting cellular targets are available for the treatment of SLE. These include biologic agents targeting B lymphocytes. However, some patients have disease refractory to these treatment modalities. For these patients, cell-based therapies may be used. Hematopoietic stem cell transplantation involving autologous cells is an option in the treatment of refractory SLE. Mesenchymal stem cells are also applied in the treatment of SLE. Chimeric antigen receptor (CAR)-T cell therapy is a novel treatment also used in SLE management. This novel treatment method holds major promise for the management of autoimmune diseases and, in particular, SLE. Major hurdles to be overcome are the logistics involved, as well as the need for specialized facilities. This review focuses on novel treatment modalities in SLE targeting cellular and molecular targets in the immune system.

## 1. Introduction

Systemic lupus erythematosus (SLE) is the prototype of systemic autoimmune diseases. The disease affects all organ systems and runs a variable course. It may run as a mild disease with periods of exacerbation and remission. It may affect critical organ systems such as the kidneys and the central nervous system. The disease may also run a very mild course. In such cases, the diagnosis and careful follow-up of the disease with the respective management is critical.

**Figure 1:**
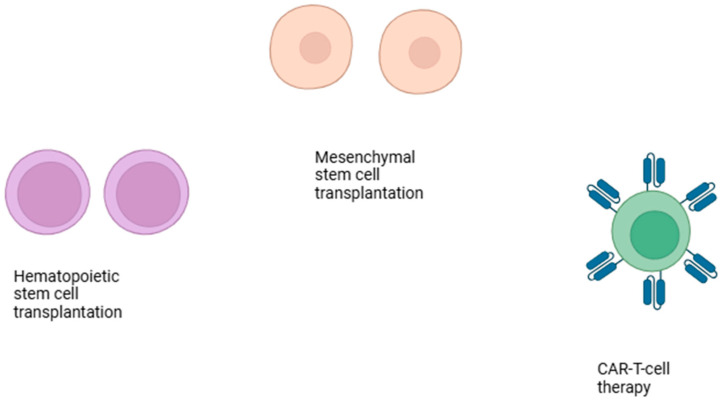
Cell-based treatment modalities for treatment of refractory systemic lupus erythematosus.

The exact etiology and pathophysiology of the disease remains elusive. It appears, however, that environmental agents act and induce the disease. Such factors are viruses and ultraviolet light, which induce the activation of the immune system and the development of an autoimmune response. Antibodies against intranuclear particles are formed, such as antinuclear antibodies, anti-ds DNA antibodies, anti-SSA (Ro) and anti-SSA (La) antibodies, and anti-Smith antibodies. Antibodies form complexes with the respective antigens and are deposited in the respective organ systems, thereby causing disease. In lupus, the clearance pathways of the organism are defective. Thus, apoptotic cells and antibody–antigen complexes are not properly cleared. In patients with severe lupus disorders of hematopoietic progenitor cells, the following have been described: increased proliferation, differentiation, and activation of cytokines and chemokines leading to differentiation toward myeloid cells [1]. Increased risk of myelodysplastic syndrome has been observed in patients with autoimmune diseases including SLE [2], indicating shared genetic susceptibility between myelodysplastic syndrome and SLE. The disease has been managed in the past through the administration of corticosteroids in various dose schedules. Various modes of treatment have been utilized over the years in cases of severe SLE. As biological drugs have emerged, a biologic agent, has been applied to the management of SLE, namely belimumab. However, recently modes of treatment and applications of modern technology have entered the field of SLE treatment (Figure 1). These treatment modalities will be discussed in this paper. In particular, hematopoietic stem cell transplantation (HSCT), mesenchymal stem cell transplantation, and CAR-T cell therapy will be discussed.

## 2. Stem Cell Transplantation

The application of modern disease-modifying modes of treatment has significantly improved outcomes in autoimmune diseases [3]. Despite these therapeutic improvements and innovations, a fraction of patients are refractory to both conventional and innovative modes of treatment [4,5]. Cure or long-term disease remission is not common [6,7]. HSCT has been applied in the management of autoimmune diseases since the last decade of the twentieth century [8,9,10,11,12,13]. Most of the cases in which it has been applied are cases of multiple sclerosis [9,14,15,16,17], systemic sclerosis [11,18,19], and Crohn’s disease [20,21,22,23], and only a minority of the cases have been of SLE [24,25,26,27,28]. It is thought that the conditioning regimen before the procedure and the subsequent infusion of the stem cells may reset the immune system as it is considered to eradicate autoreactive immune cells and allow the generation of a novel immune system that is self-tolerant [29,30]. Hematopoietic stem cell transplants, as shown in a review published in 2017, are mainly autologous [31], while allogeneic stem cell transplants are performed almost exclusively in pediatric patients. Almost two thirds of autologous stem cell transplants are delivered to patients with multiple sclerosis and systemic sclerosis. This trend is on the rise.

HSCT is a form of cellular immunotherapy [27,32,33,34]. This treatment modality involves the transfusion of hematopoietic stem cells to the recipient in order to replace the patient’s hematopoietic stem cells. Stem cell transplantation has been effectively and successfully applied in the treatment of malignant diseases [35,36]. However, it has also been applied in the treatment of autoimmune conditions. Based on the fact that SLE is a disease characterized by disorders of stem cells [37], stem cell transplantation has been applied in the treatment of SLE in cases of severe or treatment refractory disease. Stem cell transplantation is a procedure performed in multiple steps. These steps include the collection of stem cells and conditioning of the recipient with a proper regimen followed by infusion of the stem cell transplant [38]. The aim is the creation of a novel hematopoietic and a novel immune system.

HSCT has been applied in patients with hematologic diseases [35]. Early observations of remission of concurrent autoimmune disease in patients with hematologic diseases undergoing HSCT led to its application in patients with severe autoimmune diseases [39,40,41]. HSCT has been applied in patients with multiple sclerosis. It involves immunoablative treatment followed by autologous HSCT and has been found to have positive results [9]. Autologous HSCT has also been applied in patients with systemic sclerosis and has been found to improve lung function in patients with systemic sclerosis [42,43]. It has been suggested that autologous stem cell transplantation might be an option for progressive systemic sclerosis if major organ failure is imminent. Crohn’s disease has also been treated with autologous stem cell transplantation, and it may be an option for treatment-resistant disease [44].

HSCT has been used in patients with SLE [24]. Patients with disease refractory to standard and biologic treatment are candidates for this form of treatment. In a review article published in 2017, the use of hematopoietic stem cell transplantation was reviewed [31]. Hematopoietic stem cell transplantation has been applied in 279 patients with SLE, including 54 patients who also fulfilled the criteria of antiphospholipid syndrome. In the majority of the studies, an improvement in disease control as assessed with the SLEDAI (SLE Disease Activity Index) or in time free from disease was noted. In one of the studies included in the abovementioned review, no net benefit was found from HSCT compared to immunosuppression. In five patients who also had antiphospholipid syndrome, antiphospholipid antibodies were negative after stem cell transplantation, while 73% of the patients with SLE and antiphospholipid syndrome were able to discontinue coagulation. Infections were observed in 30.8% of the patients who were subjected to HSCT, while three patients succumbed to the infection. An annual incidence of infections of 11.9% was observed in the SLE patients subjected to hematopoietic stem cell transplantation. Autologous HSCT has been complicated by the appearance of aplastic anemia [45]. Secondary autoimmune diseases may also complicate HSCT [46,47,48]. Infections are a major adverse effect of HSCT [49].

Allogeneic stem cell transplantation in SLE has been fraught with adverse effects, and it may be reserved only for patients with concurrent malignant disease [26]. Autologous stem cell transplantation has been explored in a large trial with a group of 339 patients. In this trial, a disease-free survival of 50–60% at 5 years was observed [50]. Relapse risk increased with longer follow-up. The conditioning regimen before transplantation has been shown to affect the rate of remission, as a conditioning regimen of cyclophosphamide, thymoglobulin, and rituximab is related with a better remission rate [50].

HSCT is an option in the treatment of SLE, in severe cases refractory to standard treatment regimens. However, the experience so far has shown that there are major difficulties to be overcome before it enters widespread clinical practice. HSCT is characterized by mortality related to the transplant procedure and in long-term follow-up with relapse of the underlying disease. In a study [51], HSCT was related to relapse in one-third of the recipients and mortality in more than 10% related to transplantation. In a study in which hematopoietic stem cell transplantation was performed to treat lupus nephritis, a mortality of 5% was noted. The disease-free survival at 5 years was 53% and the rate of relapse was 27% [52]. As noted above, infections may occur, including cytomegalovirus infection, bacterial and/or fungal infections, allergic reactions, bone pain, and heart failure. The secondary emergence of autoimmune diseases is also a problem to be expected [48,53].

## 3. Mesenchymal Stem Cell Transplantation

Mesenchymal stem cells are adult stem cells that harbor the innate ability to self-renew and further differentiate into various types of cells. Mesenchymal stem cell treatment has been described as an option for the management of various diseases of autoimmune etiology. Such diseases, amongst others, are rheumatoid arthritis, type 1 diabetes mellitus, and multiple sclerosis [54]. The application of mesenchymal stem cell transplantations as a treatment for SLE has been investigated [55,56,57].

Sources for mesenchymal stem cells include bone marrow, umbilical cord, and adipose tissue. The procedure involves the isolation of mesenchymal stem cells, cell expansion, and infusion in the patient. Prior chemotherapy is not required. The availability of stem cells, the low rejection rate, and the absence of necessity for prior chemotherapy are advantages of this treatment modality for SLE patients refractory to standard treatment. Mesenchymal stem cells, when transplanted, regulate adaptive and immune response. The cells may downregulate inflammation and alleviate autoimmunity [58]. Findings from various studies suggest that mesenchymal stem cell transplantation is safe and has shown encouraging results as far as disease activity is concerned. However, it is not a curative option [59]. Allogeneic mesenchymal stem cell transplantation has been applied in 15 patients with active refractory SLE [56]. The patients were followed-up with for a period of up to 24 months. Severe adverse events were not noted. Disease remission was observed in this cohort, and SLE disease activity, anti-dsDNA antibodies, and proteinuria decreased. However, a relapse in proteinuria was observed in two of the patients in further follow-up. In a study involving follow-up for 6 years, it was found that allogeneic mesenchymal cell transplantation performed for refractory SLE was well tolerated by the patients and led to a decreased SLEDAI, decreased autoantibody levels, and decreased proteinuria [60]. The increased risk of infection and tumor formation was not noted. In a retrospective study, mesenchymal cell transplantation mortality was only 0.2% [61]. Mesenchymal stem cell transplantation is performed with allogeneic mesenchymal stem cells and is not yet standardized.

## 4. CAR-T Cell Therapy

Chimeric antigen receptor T (CAR-T) cell therapy is a form of technologically advanced treatment that has been applied successfully for the treatment of different types of B cell hematologic neoplasms. It was suggested that it might also be applied for the treatment of severe autoimmune disease. SLE is managed through the administration of various agents targeting B lymphocytes. However, cases of severe disease not responding to treatment or cases with severe adverse effects to this type of treatment exist. In these cases, it was thought that chimeric antigen receptor T cell therapy might be applied.

B cells are critical for the defense of the immune system against pathogens through various mechanisms, which include antibody production, the handling of antigen presentation, T cell activation and subsequent differentiation, and the production of cytokines [62]. B lymphocytes have an antigen receptor, the B cell receptor. Once the B cell receptor recognizes an antigen, the B cell is activated and undergoes proliferation and subsequent differentiation, leading to the secretion of specific antibodies [63]. B cells with autoreactive properties undergo a process of regulation during early development, leading to central tolerance and a process of regulation during later stages of maturation in peripheral lymphoid organs, leading to peripheral tolerance. A disorder in central tolerance leads to the development of autoimmune and some immunodeficiency disorders [63]. Autoimmune disease evolves when affected individuals develop aberrant T and/or B cell responses against self-proteins. It is hypothesized that responses are targeted to single immunogenic epitopes on the self-proteins. Data from animal models of autoimmunity show that the targets of immune responses in autoimmune phenomena may not remain fixed but can be extended to include cryptic epitopes on the same protein or other proteins. This procedure is called epitope spreading [64]. In the case of tissue damage, epitope spreading also occurs when tissue damage from a primary inflammatory process causes the release and exposure of a previously cryptic antigen, leading to a secondary autoimmune response against the newly released antigen. B cell epitope spreading may be involved in the pathogenesis and progression of SLE [64,65,66]. Cell surface markers such as CD19 and CD20 are expressed on B cells depending on the stage of maturation, CD19 observed on B cells from the stage of pre-B cell to plasmablast [62,67].

**Figure 2:**
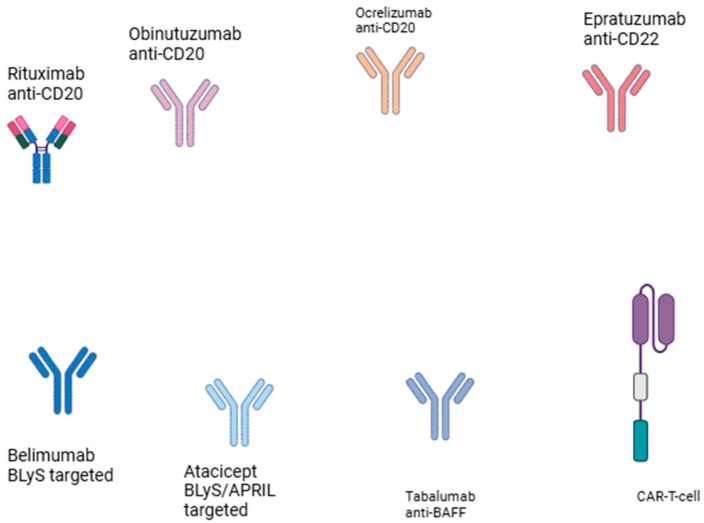
B cell-targeted treatment modalities in systemic lupus erythematosus.

**Figure 3:**
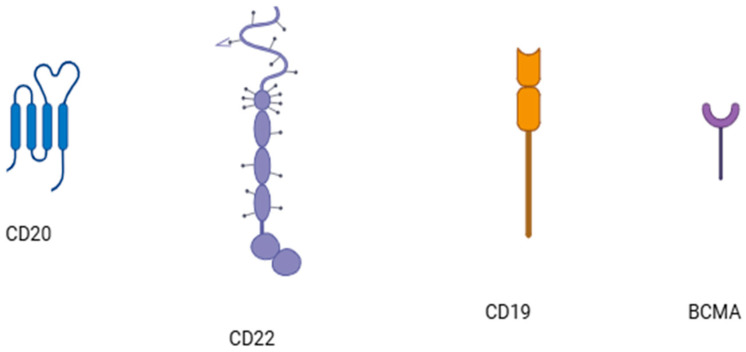
Molecular targets on B lymphocytes.

B lymphocytes are critically involved in SLE pathogenesis. Epstein–Barr virus has been implicated in the pathogenesis of SLE [68] and the virus infects B lymphocytes, where its genome may persist as an episome and may shift between a latent and a lytic phase [69]. Hence, modes of treatment targeting B lymphocytes (Figure 2) have shown beneficial effects in the treatment of SLE [70]. Modes of treatment targeting B cells utilize either the inhibition of B cells via the blockade of BAFF (B cell-activating factor) and APRIL (a proliferation-inducing ligand) [71] and B cell depletion through the application of monoclonal antibodies against B cell surface molecules, namely CD19, CD20, or CD22 [72,73]. Rituximab, an anti-CD20 monoclonal antibody; ocrelizumab, another anti-CD20 monoclonal antibody; obinutuzumab, a fully humanized anti-CD20 monoclonal antibody; and epratuzumab, a recombinant humanized anti-CD22 monoclonal antibody, have been used in lupus treatment with varied success. Rituximab induces B cell depletion via the CD20 molecule [74]. However, tissue resident B cells [75] as well as cells not expressing the CD20 molecule evade depletion, leading to incomplete response to treatment [76,77]. Ocrelizumab has been administered in lupus nephritis with partial success; however, its administration led to serious infections [78]. Obinutuzumab has been administered to patients with lupus [79], renal and non-renal patients, patients unresponsive to second-line rituximab, and patients with lupus nephritis [80]. Epratuzumab, a recombinant monoclonal antibody targeting the CD22 molecule (Figure 3) on B cells, has also been administered to lupus patients [81]. Belimumab was the first biologic agent approved for the treatment of SLE [82]. It inhibits BAFF, which is important for B lymphocyte survival [83]. Belimumab improved disease activity and flare rates [84] and was effective in renal lupus [85]. Tabalumab and blisibimod are BAFF inhibitors that have also been applied in lupus [86,87,88]. Atacicept, which aims to achieve the inhibition of both APRIL and BAFF, has also been applied in lupus cases with efficacy and no serious adverse effects [89,90,91]. Bispecific monoclonal antibodies are also applied in the treatment of SLE [28]. Cases refractory to this type of treatment exist as well as cases that respond exhibiting adverse effects.

The therapeutic approaches applied so far suggest that targeting the B lymphocyte is a fruitful approach in the management of SLE. In addition, this approach has so far been fraught with the emergence of refractory cases as well as adverse effects. Thus, novel methods targeting the B lymphocyte in the treatment of SLE have been investigated. The successful application of chimeric antigen receptor (CAR) T lymphocytes in the treatment of B cell lymphomas has led to the observation that coexistent autoimmune diseases improved [92,93,94]. Thus, the application of CAR T lymphocytes engineered to target the CD19 molecule on B lymphocytes was initiated. The aim was to target the CD19 molecule on the B lymphocyte in SLE patients and the subsequent depletion of B lymphocytes, suppressing the autoimmune process and disease remission. The application of anti-CD19 CAR T lymphocytes in a murine model of SLE indicated that this treatment modality had a preventive as well as a therapeutic efficacy as far as SLE was concerned [95]. The anti-CD19 CAR T cell treatment was administered to a female patient with active lupus nephritis refractory to treatment. The treatment led to seroconversion, i.e., anti-dsDNA antibodies were negative post treatment, and complement levels increased to normality [96]. Similar results were obtained by Taubmann et al. [97]. In a larger trial involving five patients, four female and one male, with severe refractory SLE, Mackensen et al. [98] administered anti-CD19 CAR T lymphocytes. B cell depletion occurred in all patients following treatment along with drug free remission in all eight patients. The B cell population re-emerged in the course of time following treatment. However, the re-emerging B cell population had a different non-pathogenic phenotype, indicating an immune system reset [98]. In another series of SLE patients, the administration of chimeric antigen receptor T cell treatment led to disease remission [99]. A disease activity index (SLEDAI) of 0 was observed following treatment in the lupus cohort. Anti-CD19 CAR -T cell therapy was administered to a 15-year-old female patient with lupus nephritis who was on haemodialysis [100]. The patient improved remarkably as creatinine levels decreased to normal, the glomerular filtration rate increased, proteinuria improved, and seroconversion was observed. The patient was not in need of hemodialysis following CAR-T cell treatment, and anti-hypertensive treatment was withdrawn. A double-target CAR T cell infusion harboring both BCMA (B cell maturation antigen) and CD19 molecules on CD19 B cells and plasma cells with BCMA surface antigen has been applied in patients with SLE and lupus nephritis in an open-label clinical trial [101]. The severity of lupus nephritis is related to an increased expression of BCMA in plasma cells with a long half-life [102,103]. Two patients suffering both from SLE and lymphoma achieved medication-free remission [101]. A group of nine patients suffering from lupus nephritis had symptom- and medication-free remission with a follow-up post infusion of up to 46 months. Complement levels increased to normal, and renal function and SLE disease activity index improved. Treatment was well tolerated, and the cytokine release syndrome observed was mild. B cell receptor deep sequencing was performed post infusion, revealing a complete immune reset. Through the use of specific molecular methods, it has been further shown that selective B cell depletion via CAR-T cell therapy reduces the interferon signature in SLE [104]. CAAR-T cell therapy is a further adaptation of CAR-T cell therapy that aims to deplete B cells producing specific sets of pathogenic antibodies and is now being further tested in neuroimmunology [105]. CAR-T cell therapy is accompanied by deep B cell depletion as the infused cells act autonomously, as opposed to monoclonal antibodies against B cells, which require natural killer cells, macrophages, or the complement to achieve their goal [74]. CAR therapy with alternative cells such as natural killer cells or macrophages is being evaluated [106,107].

CAR T cell therapy is a novel method, initially applied successfully in patients with B cell lymphoma and leukemia [108,109,110,111]. The treatment is accompanied by toxicity, including cytokine release syndrome (CRS) or, alternatively, cytokine-associated cytotoxicity [112], immune effector cell-associated neurotoxicity syndrome (ICANS) [113], anemia, leukopenia, thrombocytopenia [114], immunogenicity leading to anaphylaxis [115], and oncogenesis [116]. CRS is an inflammatory response that results from the activation of T lymphocytes. It complicates CAR-T cell therapy in a proportion of 42% to 93% of patients subjected to this type of treatment. It is an inflammatory response resulting from the activation of T lymphocytes and the release of IL-6 [112]. CRS is considered a consequence of the efficacy of CAR T cell infusion; however, it may be associated with undesirable outcomes [117,118,119]. CRS may manifest itself in the initial 1 to 4 days of CAR-T cell administration and may vary in severity. Severe episodes tend to occur earlier after the infusion. The levels of laboratory indicators of the acute inflammatory response, such as of C-reactive protein and ferritin, are elevated in parallel with cytokine levels, including those of IL-6 and IFN-γ [120]. CRS may vary in severity from mild, with only fever and myalgia, to severe, manifesting with cardiorespiratory dysfunction [121]. CRS has been classified into five levels of severity [117]. CRS may need only symptomatic treatment. However, more severe cases may require the administration of tocilizumab to manage [122,123]. ICANS is another complication of CAR-T cell therapy and may follow CRS, manifesting with delirium, seizures, and aphasia. Its severity is not related to CRS severity. Corticosteroids are the best treatment modality for ICANS in the context of CAR-T cell treatment [119]. Immunogenicity leading to allergic reactions may also be observed [115,124]. CAR-T cells are generated through the genomic integration of a viral vector into the genome of the recipient. Therefore, long-term oncogenicity is a concern leading to the necessity of long-term follow-up of the recipient for any malignancy. Secondary malignancies after CAR-T cell treatment have been described [116,125].

CAR-T cell therapy may be autologous or heterologous, meaning that the infused cell line may be derived from the patient’s own T lymphocytes or from the lymphocytes of an unrelated donor. Autologous CAR-T cell therapy can avoid the adversity of immunological rejection, but it requires a lengthy production, and this may be critical in severely ill patients. However, the adverse effects of host versus graft and graft versus host reactions may be avoided [126]. CAR-T cell therapy involving a rapid manufacturing protocol has been applied successfully to patients with SLE [127,128]. Sequential lymph node biopsy performed before and after CD-19 CAR T cell therapy in patients with autoimmune rheumatic diseases, including a group of patients with SLE, indicated complete B cell depletion in the lymph nodes, while T cells, macrophages, and plasma cells remained intact [129].

Treatment with CAR T cells offers a possibility of lengthy sustained remission in cases of SLE refractory to conventional modes of treatment such as treatment with biological agents or monoclonal antibodies targeting B lymphocytes. This treatment modality is characterized by cumbersome logistics, requires specialized facilities, and is accompanied by adverse effects such as cytokine release syndrome. In addition, CAR T cells are not readily available and require quite a lengthy period of production of about 4 weeks, as opposed to monocolonal antibodies or biological agents, which are ready to be administered. It should be noted that protocols with shorter production periods as well as allogeneic CAR T cell products have been tested [130]. CAR T cell therapy is investigated because it holds the potential to be a one-stop therapeutic procedure to induce permanent remission in SLE patients, refractory to standard treatment modalities. Thus, CD-19-targeted CAR-T cells from a brave new world [131] may be the future in the treatment of refractory lupus [132].

## 5. Conclusions

The prototype systemic autoimmune disease SLE may be considered a stem cell disease, and B lymphocytes are critically involved in its pathogenesis. Various drugs are available for the treatment of SLE. These include immunomodulating agents, corticosteroids, monoclonal antibodies targeting B lymphocytes, and biological agents. However, cases refractory to these treatment modalities as well as adverse effects necessitate the evolution of alternative modes of treatment. Nowadays, there are alternative treatment modalities for treating refractory lupus. These include HSCT, mesenchymal cell transplantation, and CAR-T cell therapy. Autologous HSCT has been the procedure of choice in patients with SLE, with allogeneic stem cell transplantation being reserved only for pediatric cases. Remission and, in some cases, long-term remission have been achieved following stem cell transplantation. However, infections, which are in some cases fatal, have emerged. In addition, secondary autoimmune phenomena complicate the procedure. Mesenchymal cell transplantation is another stem cell procedure that has been tested in SLE. The procedure of choice has been allogeneic mesenchymal cell transplantation. However, the procedure has not entered clinical practice successfully. CAR-T cell therapy was applied successfully in patients with hematologic malignancies such as leukemia, lymphoma, and myeloma. CAR-T cell therapy targeting CD-19 on B lymphocytes proved successful in cases with hematologic malignancies. In addition, it was noted that autoimmune conditions went into remission. As B cell-targeted monoclonal antibodies proved therapeutically successful, targeting the B lymphocyte with CD-19-targeted CAR-T cells has been a promising approach for the treatment of SLE patients refractory to standard treatment. Thus, CD-19-targeted CAR T cell therapy has been administered to patients with SLE. The procedure was followed by long-lasting remission, and adverse events such as cytokine release syndrome and immune effector cell neurotoxicity syndrome were manageable. CAR-T cell therapy with alternative targets as well as CAR therapy with alternative cell types are being investigated. CAR-T cell therapy with preparations for ready administration to recipients is also under research in an effort to provide a readily prepared off-the-shelf treatment.

In conclusion, cell therapy is an option for SLE refractory to standard treatment. CAR-T cell therapy holds a major promise for the achievement of sustained remission over older methods such as hematopoietic stem cell transplantation or mesenchymal cell transplantation.

## Data Availability

No new data were created or analyzed in this study. Data sharing is not applicable to this article.
